# Sustainable healthcare in medical education: survey of the student perspectives at a UK medical school

**DOI:** 10.1186/s12909-022-03737-5

**Published:** 2022-09-23

**Authors:** Dhruv Gupta, Lahvanya Shantharam, Bridget K MacDonald

**Affiliations:** 1 St George’s University of London, Cranmer Terrace, Tooting, London, SW17 0RE UK; 2 St George’s University Hospitals NHS Foundation Trust, Blackshaw Road, London, SW17 0QT UK

**Keywords:** Education for sustainable healthcare, Medical education, Sustainable healthcare

## Abstract

**Background:**

It is now a General Medical Council requirement to incorporate education for sustainable healthcare (ESH) into medical curricula. To date, research has focussed on the perspectives of educators and which sustainable healthcare topics to include in teaching. Therefore, due to this gap in the literature, we have investigated the perspectives of medical students in the UK regarding current and future incorporation of ESH in medical education.

**Methods:**

A survey was circulated to 851 clinical year medical students and students intercalating after completing at least one clinical year in a London University. The anonymous survey consisted of sections on the environmental impact, current teaching and future teaching of ESH.

**Results:**

One hundred sixty-three students completed the survey. 93% of participants believed that climate change is a concern in current society, and only 1.8% thought they have been formally taught what sustainable healthcare is. No participants strongly agreed, and only 5 participants (3.1%) agreed, that they would feel confident in answering exam questions on this topic, with 89% agreeing that more ESH is needed. 60% believe that future teaching should be incorporated in both preclinical and clinical years, with 31% of participants preferring online modules as the method of teaching.

**Conclusion:**

Our study has stressed the lack of current sustainable healthcare teaching in the medical curriculum. There is student demand for ESH, however, uncertainty remains regarding who is best placed to facilitate ESH, how it should be delivered, and whether there is a gender discrepancy regarding sustainable healthcare importance, emphasising the need to close the gap between educational rhetoric and action.

## Background

Climate change plays an increasingly significant role in our daily lives. From a medical perspective, air pollution is associated with 31 adverse health outcomes, including cancer and stroke [1, 2]. “Climate change is the greatest global health threat of the 21st century” [3]. Poor air quality is a significant cause of annual mortality, with air pollution causing 7 million deaths globally in 2018 [4, 5], and it is predicted that there will be 1 billion climate migrants due to climate change by 2050 [6]. Therefore, it is becoming increasingly evident that climate change is adversely impacting health outcomes internationally.

The healthcare sector has a significant environmental impact, with the UK’s National Health Service (NHS) having the largest carbon footprint in the UK public sector [7], and the healthcare sector contributing to 4.4% net global emissions worldwide [8]. Therefore, healthcare services have an increasingly recognised responsibility to become sustainable, with 52 countries committing to develop lean, low-carbon, and climate-resilient healthcare services during COP26 [9]. Sustainable healthcare can be defined as “education about the impact of climate change and ecosystem alterations on health, and the impact of the healthcare system on the aforementioned” [10].

The COVID-19 pandemic has placed environmental concern at the forefront of debate, with the majority of the UK public sampled in polls believing that governmental action should prioritise environmental protection post-pandemic [11]. The pandemic has been both beneficial and detrimental to the environment: positives are associated with lockdowns held internationally and reduced economic activity, whereas negatives are associated with reduced recycling and increased demand for personal protective equipment (PPE) [12]. PPE is particularly pertinent for sustainable healthcare: the World Health Organisation (WHO) has estimated that 89 million medical masks have been needed each month during the COVID-19 pandemic. It has been suggested that the COVID-19 pandemic could be used to introduce environmental and economic policies that can benefit long-term health outcomes [13, 14]. Therefore, while undoubtedly a human tragedy, the COVID-19 pandemic has reiterated the importance of sustainable economic recovery and healthcare for current and future generations.

Alongside current healthcare professionals, healthcare professionals in training are arguably best placed to be educated about sustainable healthcare relevant for current and future generations. The AMEE Consensus Statement emphasises the roles that universities have in developing healthcare students to appropriately face the problems of climate change and promote sustainability [15]. From a UK perspective, the General Medical Council (GMC) have stated in Subsection 25 of their Outcomes for Graduates that newly qualified doctors should understand and be able to utilise principles of sustainable healthcare in their medical practice, and that universities had until 2020 to put in the necessary teaching to support this   [16, 17]. However, the Planetary Health Report Card (PHRC) suggests that education for sustainable healthcare (ESH) may still be lacking internationally [18]. Therefore, it appears clear that sustainable healthcare in medical education is still in its infancy, with relatively little published thus far [19, 20].

Tun’s research gained the perspective of educators regarding the integration of ESH in medical education [21]. Several obstacles were identified including the perceived lack of teaching time, and a notable enabler included student interest in sustainable healthcare. However, this did not consider the perspective of current medical students. She also identified a concern that medical educators may not be sufficiently informed to teach students well [21]. To address this, the use of peer teaching from fellow medical students has been proposed by Green and Legard. While they summarised the views of several medical schools, this included a relatively small cohort of 29 medical students [22]. Additionally, Teherani et al. surveyed 52 sustainable healthcare experts, who identified that most teaching should be undertaken in preclinical years [19].

There does not seem to be a clear consensus on how to best integrate sustainable healthcare into medical curricula. This lack of clarity may be explained by the fact that, to our knowledge, no previous study has investigated the perspective of current medical students in literature regarding current and future incorporation of sustainable healthcare in medical education. Therefore, gaining insight of the students’ perspective on sustainable healthcare will enable us to identify pitfalls in current medical education, and approaches to maximise the efficacy of ESH in the future. We aim to identify:

- Whether current medical students have been taught what sustainable healthcare is.
- Whether current ESH is sufficiently incorporated into the medical curriculum.
- The importance of sustainable healthcare from the perspective of current medical students.
- Preferred approaches to include ESH in the medical curriculum.

## Methods

### Participants

Inclusion criteria for choosing participants for this study involved current medical students in clinical years at a London university (years 3, 4 or 5 respectively) or students currently intercalating having completed at least one clinical year. Prospective students were invited via central emails and social media to complete the survey, which was open over a 2 week period.

### Materials and design

The anonymous survey consisted of four sections: demographics, environmental impact, current teaching and future teaching. Demographic data collected included gender and year of study in medical school. Questions asked regarding environmental impact, current teaching, and future teaching are summarised in Tables 1 and 2, and Figs. 2 and 3, and cover the overarching principles of the priority learning outcomes from the Centre for Sustainable Healthcare [23]. Environmental impact and current teaching sections of the survey were assessed via a Likert scale. Microsoft Forms was used to design the survey and interpret the data collected. Microsoft Excel was also used to interpret the data. All questions had to be completed for the participant to be able to submit the survey.

**Table 1:** Assessing the student perspective on environmental impact in relation to both current society as well as medical practice via Likert scale. Percentages to 2 significant figures

Statement	Strongly disagree	Disagree	Neither agree nor disagree	Agree	Strongly agree
*I believe that climate change is a significant concern in current society.*	1 (0.6%)	2 (1.2%)	9 (5.5%)	47 (29%)	104 (64%)
*I am conscious of my daily impact on the environment.*	1 (0.6%)	20 (12%)	19 (12%)	88 (54%)	35 (22%)
*Daily medical practice adversely impacts the environment.*	0 (0.0%)	6 (3.7%)	39 (24%)	82 (50%)	36 (22%)
*It is important for daily medical practice to be environmentally friendly.*	2 (1.2%)	3 (1.8%)	18 (11%)	70 (43%)	70 (43%)
*HCP’s should consider their impact on the environment in daily practice.*	2 (1.2%)	8 (4.9%)	16 (9.8%)	78 (48%)	59 (36%)

**Table 2:** Assessing the student perspective on current sustainable healthcare teaching in the medical curriculum. Percentages to 2 significant figures

Statement	Strongly disagree	Disagree	Neither agree nor disagree	Agree	Strongly agree
*My course has made it clear that it is a GMC requirement for newly qualified doctors to understand and utilise the principles of sustainable healthcare in their medical practice.*	51 (31%)	79 (48%)	17 (10%)	13 (8.0%)	3 (1.8%)
*I have been formally taught what sustainable healthcare is. This can be defined as “education about the impact of climate change and ecosystem alterations on health, and the impact of the healthcare system on the aforementioned” (CSH Networks).*	84 (52%)	63 (39%)	13 (8.0%)	2 (1.2%)	1 (0.6%)
*I have been formally taught about environmentally friendly plans already established in the NHS.*	86 (53%)	64 (39%)	9 (5.5%)	4 (2.4%)	0 (0.0%)
*I would feel confident in answering questions about sustainable healthcare in an exam setting.*	104 (64%)	46 (28%)	8 (4.9%)	5 (3.1%)	0 (0.0%)
*More teaching is needed about sustainable healthcare in the medical curriculum.*	2 (1.2%)	5 (3.1%)	10 (6.1%)	51 (31%)	95 (58%)
*There is time and space available in the curriculum to incorporate teaching about sustainable healthcare.*	9 (5.5%)	24 (15%)	51 (31%)	60 (37%)	19 (12%)

## Results

### Demographics

**Fig. 1:**
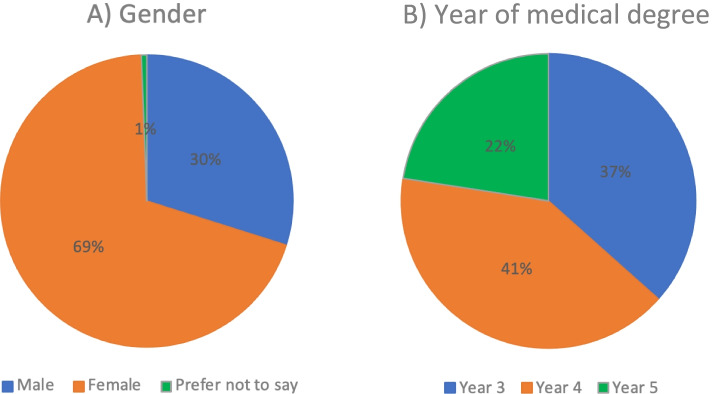
Demographics of respondents. **A** Gender of all respondents. **B** Year of study. Respondents intercalating were asked to choose their last clinical year completed

One hundred sixty-three medical students completed the survey out of a possible 851 students. Demographics are summarised in Fig. 1.

### Environmental impact

Answers to statements on environmental impact in relation to both current society as well as medical practice are summarised in Table 1.

### Current teaching

Answers to statements on current ESH in the medical curriculum are summarised in Table 2.

### Future teaching

When asked how sustainable healthcare should be incorporated into teaching, respondents voted for the following: 50 for online modules (31%), 42 for lectures (26%), 39 for small group teaching (24%), 26 for ward-based teaching (16%), and 6 for ‘other’ (3.7%). For ‘other’ responses, students specified that teaching should be as ‘1-2 lectures online’, it should be ‘integral to part of all teaching rather than seen as an extra’, ‘ward-based teaching’, ‘don’t know’, ‘a combination of small groups, lectures, and ward-based learning’, and ‘all of the above are useful’.

Forty-one students (25%) voted that teaching should be incorporated in preclinical years, 24 for clinical years (15%), 97 for both preclinical and clinical years (60%), and 1 for other (0.6%), with the latter specified as ‘all stages as spiral learning’.

**Fig. 2:**
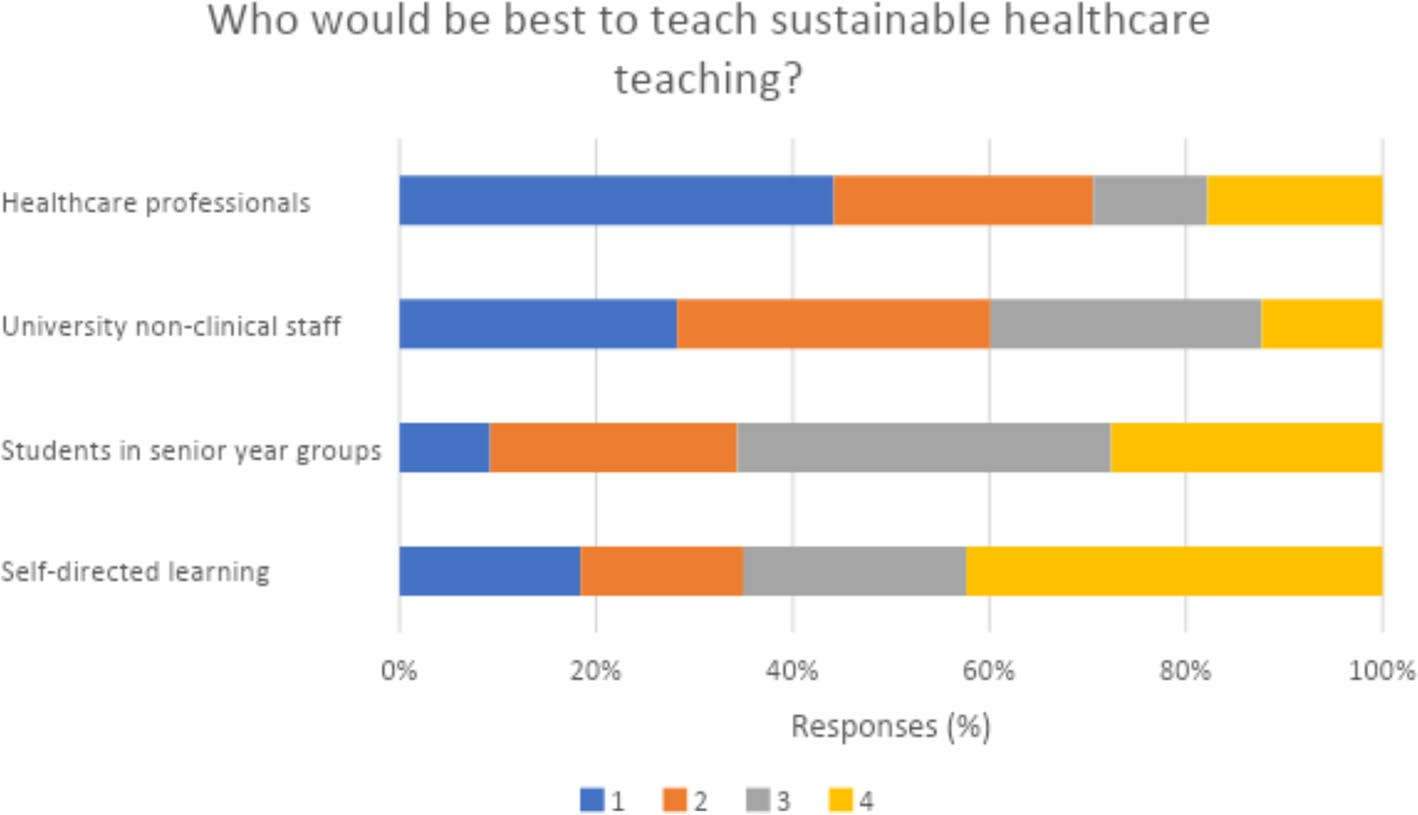
Student perspective ranking who would be best to teach sustainable healthcare. 1 = most appropriate, 4 = least appropriate

**Fig. 3:**
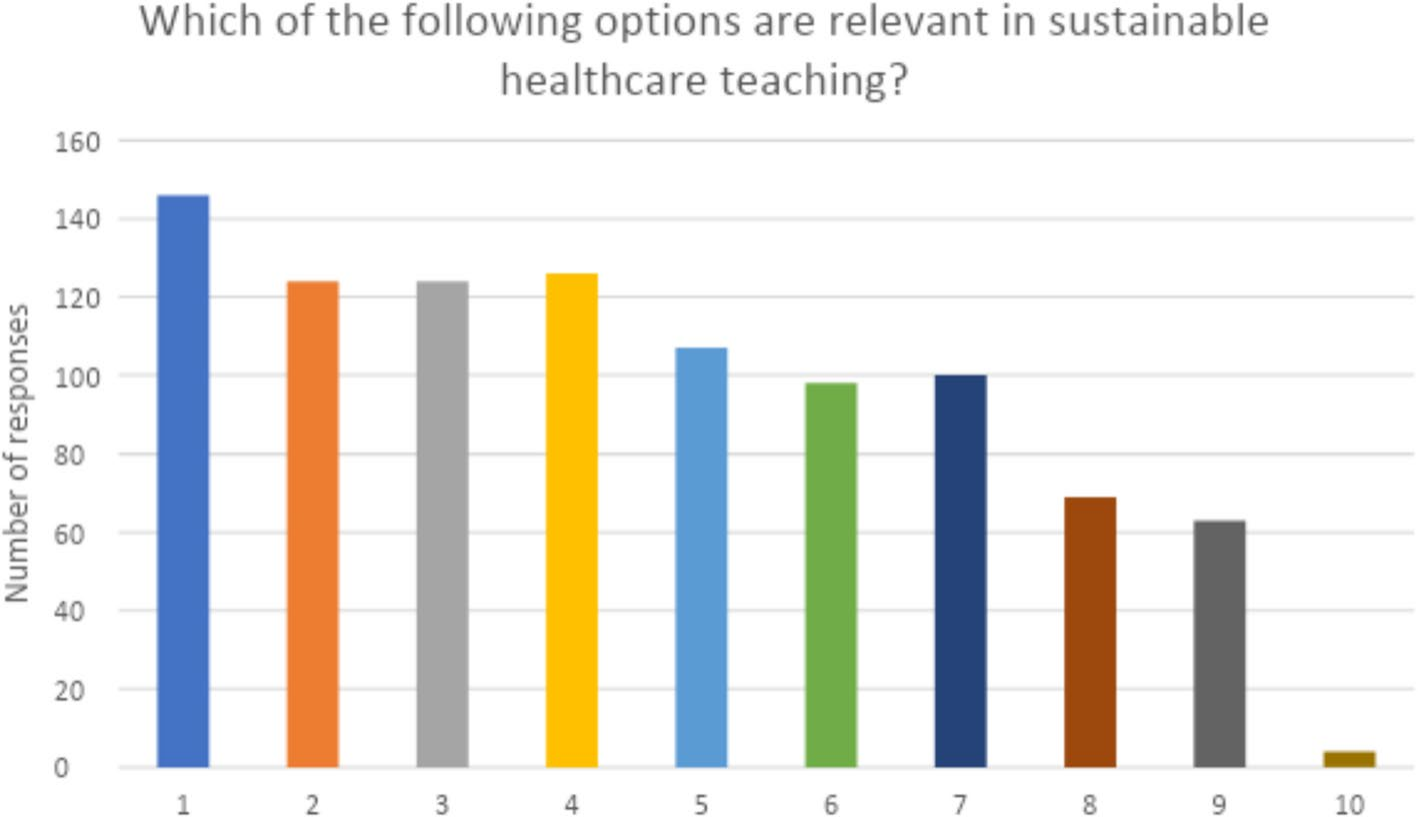
Student opinion on what topics would be relevant to teach about sustainable healthcare in medical curricula. X-axis abbreviations: 1 = the NHS’ impact on climate change, 2 = the effect of climate change on medical conditions, 3 = already established approaches to reduce the environmental impact of the NHS (e.g. the NHS Long Term Plan), 4 = new approaches to reduce the environmental impact of the NHS, 5 = how COVID-19 has made us re-think strategies to implement healthcare, 6 = renewable energy resources and their utility in healthcare, 7 = financing sustainable healthcare and pitfalls to overcome, 8 = relevant research from other industries, 9 = additional resources available regarding the environmental impact of medical practice, 10 = other

Figure 2 summarises student responses when asked who would be best to give this teaching, and Fig. 3 summarises potential sustainable healthcare topics of student interest to be included in future teaching.

## Discussion

### Demographics

One hundred sixty-three responses were received from a potential 851 respondents (19%). This relatively low response rate is comparable to a similar Yale study amongst US healthcare students, with possible explanations including selection bias: students with prior interest are more likely to respond to the survey [24]. An additional reason may be that the survey was released shortly after hospital placements had restarted for clinical year medical students at the London university after initial lockdowns held in the UK due to the COVID-19 pandemic. Therefore, the timing of the survey release may have further hindered the response rate.

69% of respondents were female. Table 3 displays the differences in responses to the survey based on gender. Results of note include a greater proportion of females felt that it is important for daily medical practice to be environmentally friendly (89%), that healthcare professionals should consider their impact on the environment in daily practice (90%), that the course had not made it clear that ESH is a GMC requirement (85%), and that there is time and space in curriculum to incorporate ESH (53%), in comparison to males (78, 72, 65, 37% respectively). While we are unable to comment on whether these gender discrepancies are statistically significant, this appears to be in keeping with the Yale study, where more females regarded sustainable healthcare as an important issue compared to males [24]. There is limited evidence currently available regarding a possible gender discrepancy between sustainable healthcare importance. Therefore, future research should identify whether this is statistically significant, possible drivers, and whether ESH should be tailored to ameliorate this discrepancy.

**Table 3:** Sub-demographic data assessing responses based on gender via Likert scale. Percentages up to 2 significant figures

Statement	Gender	Strongly agree	Agree	Neither agree nor disagree	Disagree	Strongly disagree
*I believe that climate change is a significant concern in current society.*	Male	28 (57%)	15 (31%)	5 (10%)	1 (2.0%)	0
	Female	76 (67%)	32 (28%)	4 (3.5%)	1 (0.9%)	1 (0.9%)
	Prefer not to say	0	1 (100%)	0	0	0
*Daily medical practice adversely impacts the environment.*	Male	15 (31%)	19 (39%)	10 (20%)	5 (10%)	0
	Female	22 (19%)	62 (54%)	29 (25%)	1 (0.9%)	0
	Prefer not to say	0	1 (100%)	0	0	0
*It is important for daily medical practice to be environmentally friendly.*	Male	17 (35%)	21 (43%)	7 (14%)	3 (6.1%)	1 (2.0%)
	Female	53 (46%)	49 (43%)	11 (9.6%)	0	1 (0.9%)
	Prefer not to say	1 (100%)	0	0	0	0
*Healthcare professionals should consider their impact on the environment in daily practice.*	Male	17 (35%)	18 (37%)	9 (18%)	4 (8.2%)	1 (2.0%)
	Female	41 (36%)	61 (54%)	7 (6.1%)	4 (3.5%)	1 (0.9%)
	Prefer not to say	1 (100%)	0	0	0	0
*My course has made it clear that it is a GMC requirement for newly qualified doctors to understand and utilise the principles of sustainable healthcare in their medical practice.*	Male	2 (4.1%)	5 (10%)	10 (20%)	21 (43%)	11 (22%)
	Female	8 (7.0%)	8 (7.0%)	8 (7.0%)	58 (51%)	39 (34%)
	Prefer not to say	0	0	0	0	1 (100%)
*I have been formally taught what sustainable healthcare is. This can be defined as “education about the impact of climate change and ecosystem alterations on health, and the impact of the healthcare system on the aforementioned” (CSH Networks).*	Male	1 (2.0%)	1 (2.0%)	4 (8.2%)	20 (41%)	23 (47%)
	Female	0	1 (0.9%)	4 (7.9%)	44 (39%)	60 (53%)
	Prefer not to say	0	0	0	0	1 (100%)
*I have been formally taught about environmentally friendly plans already established in the NHS.*	Male	0	1 (2.0%)	5 (10%)	18 (37%)	25 (51%)
	Female	0	3 (2.6%)	4 (3.5%)	47 (41%)	60 (53%)
	Prefer not to say	0	0	0	0	1 (100%)
*I would feel confident in answering questions about sustainable healthcare in an exam setting.*	Male	0	3 (6.1%)	2 (4.1%)	17 (35%)	27 (55%)
	Female	0	2 (1.8%)	7 (6.1%)	29 (25%)	76 (67%)
	Prefer not to say	0	0	0	0	1 (100%)
*More teaching is needed about sustainable healthcare in the medical curriculum.*	Male	18 (37%)	21 (43%)	7 (14%)	4 (4.1%)	1 (2.0%)
	Female	77 (68%)	30 (26%)	3 (2.6%)	3 (2.6%)	1 (0.9%)
	Prefer not to say	1 (100%)	0	0	0	0
*There is time and space available in the curriculum to incorporate teaching about sustainable healthcare.*	Male	2 (4.1%)	16 (33%)	13 (27%)	11 (22%)	7 (14%)
	Female	16 (14%)	44 (39%)	38 (33%)	14 (12%)	2 (1.8%)
	Prefer not to say	1 (100%)	0	0	0	0

### Environmental impact

“Climate change is the biggest global health threat of the 21st century” [3]. Costello et al’s quote from 2009 fittingly resonates to this day, with global warming estimated to cause 250,000 extra deaths globally each year between 2030 and 2050 [25]. Therefore, it is unsurprising that 83% of participants agreed or strongly agreed that climate change was a significant concern in current society.

Furthermore, 86% of respondents believe that daily medical practice should be environmentally friendly, and 72% felt that daily medical practice adversely impacts the environment, in keeping with the Yale study, and reiterating the importance of the NHS’ “Delivering a ‘Net Zero’ National Health Service” by 2040 and the commitment that 52 countries made in COP26 [9, 24, 26].

### Current teaching

One of the GMC outcomes for graduates is that “newly qualified doctors must be able to apply the principles, methods and knowledge of population health and the improvement of health and sustainable healthcare to medical practice” [16]. Despite this statement, our results indicate that 79% of students did not believe that their course had made this clear. The majority of students felt they had not been formally taught what sustainable healthcare is, in keeping with El Omrani et al’s findings [27]. However, there is student demand to incorporate sustainable healthcare into medical curricula, complementing Tun’s findings from the perspective of medical educators [21].

Assessing learning is considered a barrier to introducing sustainable healthcare into medical curricula [21]. In this study, 92% of students did not feel confident in answering sustainable healthcare-related exam questions. Therefore, perhaps the more pressing issue at hand is the lack of coherent ESH, as opposed to assessing sustainable healthcare learning. Using formative but mandatory assessments has been suggested [28], and may help students to gain ESH, while transitioning to sustainable healthcare in summative examinations.

Incorporating ESH into student-selected components already embedded into medical curricula may also provide an opportunity to introduce ESH, and could be linked to quality improvement, which forms its own section in the GMC Outcomes for Graduates [16, 29]. Furthermore, if associated with a clinical supervisor, this may help to give a clinical insight into sustainable healthcare, alongside providing an opportunity for current healthcare professionals to learn about sustainable healthcare as well. Alternative methods of incorporating ESH include reflective writing, short answer questions in summative examination, and part of clinical placements [30], with the former relating to section 2 of the GMC Outcomes for Graduates: “Professional and ethical responsibilities” [16].

Methods of embedding sustainable healthcare into the curriculum has been discussed in literature internationally, with medical educators describing the curriculum as already overcrowded [21, 24, 31–33]. This study reinforces the uncertainty of whether there is sufficient space in the medical curricula for ESH, but from a student perspective. The aforementioned solutions integrate ESH into the curriculum [21], instead of removing content from the syllabus, thereby enhancing the intertwined relationship between sustainable healthcare and medical practice. It is therefore essential for further research to be conducted on how to best incorporate ESH into medical education, to urgently address this international issue.

### Future teaching

The student perspective suggests that online modules (31%) are the most popular method of incorporating sustainable healthcare into medical education. This was similar to lectures (26%) and small group teaching (24%), suggesting that uncertainty remains on how they would prefer to be taught. However, it is important not to interpret these results as generalisable beyond the study population. The response rate was too low for representative sampling of the student population, and student preference does not equate to effectiveness of a given teaching modality. The COVID-19 pandemic may have skewed this result however, as online modules were the only option which did not involve in-person interaction [34]. This uncertainty on how best to be taught sustainable healthcare is also reflected by educators, with an Australian university study finding that two-thirds of educators would not know the best way for their students to be taught [32].

Students were also asked to rank who would be best to teach sustainable healthcare. Healthcare professionals were the preferred option, followed by university non-clinical staff (45 and 28% as most appropriate choice respectively), with only 9% preferring to be taught by senior students. Tun identified that medical educators may not be able to effectively teach students due to lack of knowledge, and that students and medical educators both teaching and learning from one another may be the preferred ESH method [21]. However, our results suggest that from a student perspective, students prefer to be taught by healthcare professionals and non-clinical staff, in comparison to peer students.

The results highlight further uncertainty regarding the ideal approach to provide ESH, with similar response rates for students to be taught through online modules (30%), lectures (26%), and small group teaching (24%) respectively. It has been previously suggested that pooled resources across medical schools can help to minimise the lack of ESH available [20–22]. Our survey asked participants to rank their preferred teaching method, however, there was no clear preference with modalities scoring similarly. This may also reflect a discrepancy between educational preference and educational effectiveness. Therefore, this uncertainty surrounding how to best teach a new subject in the medical curriculum also presents the opportunity to study which teaching modalities are effective. This includes conducting further research from the perspectives of both students and medical educators to identify whether one ESH teaching method is superior to others, and by comparing ESH teaching methods both intra- and inter-institutionally.

### Strengths and limitations

This paper addresses a gap in the literature on the perspectives of medical students in the UK regarding current ESH in medical education. This study investigated the preferred method of incorporating future ESH in medical education from a student perspective. Although previous studies have asked students for opinions, the sample size for our study was larger (*n* = 163).

Despite having a large sample size compared to previous similar UK studies, we only collected data from one London medical school. Our survey did not have many ‘open questions’, meaning that students could not fully voice their opinion. Furthermore, we only distributed the survey amongst students in their clinical years, which meant we did not gain the insight of students in preclinical years. We decided not to distribute the survey to preclinical students due to their relative lack of exposure to a clinical environment. Use of a Likert scale for most of our questions meant that we did not obtain much qualitative data. Significance of our findings could also not be determined as we did not perform any statistical analysis. We used statements for the environmental impact and current teaching sections. The survey lacked qualitative exploration, and therefore, categorical choices were not necessarily complete. For example, the choice of having ESH integrated across all modules, as opposed to a stand-alone e-learning module, was not specifically included, with the closest option being to incorporate ESH into both pre-clinical and clinical years. Future iterations of this survey could address this issue. Furthermore, with the Medical Schools Council (MSC) recently releasing an ESH curriculum [35], mapping our survey to this curriculum will create a more comprehensive survey.

### Future research

Sustainable healthcare in medical education remains a relatively novel concept, with limited research thus far. Our study gained the perspective of medical students in clinical years from a London university. Distributing a modified survey reflecting the MSC curriculum, with more qualitative elements and comprehensive categorical choices, among medical schools across the UK and globally in the future will allow us to gain further insight on the student perspective of ESH. Additionally, repeating this study in the future will allow us to longitudinally compare whether ESH has improved in medical education after medical schools have had more time to incorporate and develop such teaching in their curricula. Using small focus groups may allow students to further articulate their views on how to embed sustainable healthcare into the curriculum in a more qualitative manner, and enhance ESH.

While we only focussed on the perspective of medical students in clinical years, it may prove useful to gain insight from preclinical students, who may have a less medical perspective on sustainable healthcare, and provide a more generalised approach instead. Finally, to gain an updated insight into the medical educator perspective, distributing a similar survey, but tailored to educators, across all UK medical schools will allow us to compare and contrast this to the student opinion, and identify an optimal method to implement ESH. This is particularly important where uncertainty remains in our study, such as identifying who is best placed to teach sustainable healthcare, how sustainable healthcare should be taught, and whether there is a gender discrepancy present regarding the importance of sustainable healthcare. Researching whether there is a discrepancy between ESH in the UK and globally will highlight any further improvements that should be made to our teaching. Finally, with increasing emphasis to integrate ESH into medical curricula, future research should determine whether teaching has been successfully incorporated to effectively teach students and raise awareness once they have transitioned into clinical practice.

## Conclusion

This study investigated the perspective of medical students in the UK regarding current and future incorporation of sustainable healthcare in medical education. Students believe that it is important for daily medical practice to be environmentally friendly, but it currently isn’t. Most respondents feel that they have not been formally taught what sustainable healthcare is and would not feel confident on being examined on this topic, despite it being a GMC requirement. Although respondents expressed a preference for expert rather than peer teaching, uncertainty remains on how it should be taught, and whether there is a gender discrepancy present regarding the importance of sustainable healthcare. Our research highlights a clear discrepancy between educational rhetoric and action on sustainable healthcare. Therefore, it is essential that this gap is closed if the NHS is to meet its Net Zero commitment.

## Data Availability

The datasets used and/or analysed during the current study are available from the corresponding author on reasonable request.

## Abbreviations

ESH: Education for sustainable healthcare
GMC: General Medical Council
NHS: National Health Service
PHRC: Planetary Health Report Card
PPE: Personal Protective Equipment
UK: United Kingdom
WHO: World Health Organisation
